# *NtANTL2* overexpression regulates starch-related and nitrogen metabolism in tobacco plants

**DOI:** 10.1186/s12870-025-06748-8

**Published:** 2025-06-07

**Authors:** Bo Lei, Fei Wang, Huina Zhao, Benbo Xu, Le Xu, Lingli Xie

**Affiliations:** 1 Guizhou Academy of Tobacco Science, Guiyang, 550081 Guizhou China; 2 MARA Key Laboratory of Sustainable Crop Production in the Middle Reaches of the Yangtze River (Co-Construction By Ministry and Province), College of Agriculture, Engineering Research Centre of Ecology and Agricultural Use of Wetland, Ministry of Education, Yangtze University, Jingzhou, 434025 Hebei China

**Keywords:** Tobacco, Nitrogen, *ANT1-like gene 2*

## Abstract

The function of the aromatic and neutral amino acid transporter gene ANT1-like (*NtANTL2*) was investigated. *NtANTL2-3* over-expressor plants displayed greater tolerance to high nitrogen application compared to WT plants, characterized by better growth. As for carbon metabolism, the activity of sucrose phosphate synthetase in overexpression lines was 1.5 times higher than that in wild-type plants at 0.1% nitrogen, while sucrose synthase activity was significantly lower under all nitrogen levels. These changes in enzyme activities were closely associated with the differential gene expressions of *GBSSI* (*granule—bound starcsssh synthase I*), *SUS* (*sucrose synthase*), *INV* (*invertase*), and *SPS* (*sucrose phosphate synthase*). The activities and gene expressions of key enzymes in nitrogen metabolism like nitrate reductase, glutamine synthetase, glutamate synthase, and glutamate dehydrogenase were also significantly affected. The GDH activity in overexpression lines was increased by 2.4-fold and 4.3 fold at 0.1% and 0.3% nitrogen, respectively. Increased levels of multiple amino acids besides aspartic acid content were found in *NtANTL2* overexpression plants. These results clearly indicate the regulatory ability of *NtANTL2* on amino acid composition and its central role in coordinating carbon and nitrogen metabolism balance.

**Supplementary Information:**

The online version contains supplementary material available at 10.1186/s12870-025-06748-8.

## Introduction

Flue-cured tobacco (*Nicotiana tabacum* L.) is an economically significant, non-edible field crop, widely cultivated worldwide and all over China [1]. Nitrogen (N) availability is a critical limiting factor affecting biomass production and metabolic processes [2, 3]. Consequently, to achieve higher yields and improve economic efficiency, farmers often apply substantial quantities of N fertilizers [4, 5]. However, excessive nitrogen fertilizer can make the aroma become weak or be masked by a strong nicotine smell and excessive use of N fertilizers leads to environmental pollution and soil acidification [5–8]. Enhancing nitrogen use efficiency (NUE) in flue-cured tobacco plants is a crucial strategy for mitigating the environmental pressures associated with excessive N fertilizer application [9].

In most plants, nitrogen is primarily assimilated into amino acids in roots or shoots to support plant growth [10–12]. Amino acids are involved in the tricarboxylic acid cycle, generate cellular energy [13], and regulate the carbon-to-nitrogen (C/N) balance [14]. Amino acid transporters facilitate root uptake, xylem loading in roots, phloem loading in leaves, and nitrogen import into seeds. Based on sequence similarities and uptake properties, amino acid transporter families in plants are classified into two subfamilies: amino acid/auxin permeases (AAP) and amino acid polyamine and choline transporters [15–17].

Among plant amino acid transporters, lysine histidine transporter 1 (LHT1) and AAP families and their roles in nitrogen (N) and carbon (C) metabolism, growth, and development were well studied. *Arabidopsis* *AtLHT1*, highly expressed in roots, young leaves, flowers, and leaf sheaths, mediates uptake of neutral and acidic amino acids into mesophyll cells. Overexpression of *AtLHT1* or cucumber homologs *CsLHT1*/*CsLHT6* enhances amino acid uptake, while *AtLHT1* loss reduces aboveground biomass and seed yield under N limitation [18–20]. In rice, *OsLHT1* knockout impairs root uptake of root-synthesized amino acids, decreasing N absorption efficiency and yield but increasing seed protein content and essential free amino acids [21, 22]. *NtLHT1* is a low-affinity amino acid transporter which transports neutral, acidic, non-polar, and aromatic amino acids, as well as the ethylene precursor ACC (1-aminocyclopropane-1-carboxylate), gibberellin, and indole-3-acetic acid. *NtLHT1* influences key physiological processes such as cell division, elongation, and differentiation by modulating the levels of amino acids and plant hormones [23].

The AAP family exhibits functional diversity: *AtAAP1*, localized at root tips, epidermal cells and regulated by light, C status, and nitrate, influences *Arabidopsis* growth, seed development, protein storage, and yield [24, 25]. In pea (*Pisum sativum*), overexpression of *AAP1* improved nitrogen uptake and utilization efficiency [26]. *AtAAP2* in phloem facilitates N translocation from xylem to phloem, with *aap2* mutants allocating more N to leaves to enhance photosynthesis and seed yield [27, 28]. *AtAAP3* in root vascular tissues may mediate amino acid uptake from soil or phloem, and *AtAAP5* transports cationic amino acids [29, 30]. Rice *OsAAP6* overexpression increases seed storage proteins without affecting yield [27], while *AtAAP8* in leaf phloem and petioles is critical for loading amino acids into phloem for seed allocation; *aap8* mutants show reduced phloem loading, impaired photosynthesis, and N/C assimilation [31, 32]. Disruption of rice *OsAAP3*, *OsAAP5*, or *OsAAP7*—functions contrasting with LHT1—promotes tillering and increases grain yield, highlighting complementary roles in regulatory networks [33–35].

Aromatic and neutral amino acid transporters (ANTs) belong to the amino acid/auxin permease subfamily [36], with genes like *ANTL2* capable of transporting diverse substrates to meet physiological demands [11]. In *Arabidopsis*, *AtANT1* mediates the transport of aromatic and neutral amino acids, including arginine, exhibiting tissue-specific expression modulated by nutrient status and environmental cues. For instance, seedling *AtANT1* expression is induced by varying nitrate concentrations, linking its activity to nitrogen signaling and amino acid homeostasis [37]. Wheat *TaANT1* shows peak expression in endosperm aleurone cells 20 days post-anthesis, suggesting a role in delivering N to developing grains during the mid-filling stage. Genetic analysis in *Arabidopsis* reveals that the *ant1* mutant accumulates more free amino acids in sieve tubes significantly than wild-type plants, indicating that ANT1 facilitates amino acid removal from the phloem, likely through involvement in phloem unloading [38, 39].

This function underscores ANT1’s role in regulating source-to-sink amino acid partitioning, where disrupted transport alters long-distance N distribution and metabolic balance. Collectively, these findings highlight ANTs as key mediators of amino acid transport, environmental responses, and nutrient allocation across plant tissues, with implications for understanding N metabolism and optimizing crop productivity. Although there are few detailed studies directly on ANT proteins in tobacco, similar regulatory mechanisms are likely present in tobacco, suggesting that these transporters are finely tuned to meet the plant's metabolic needs [40].

The amount and form of available nitrogen significantly affect plant growth, photosynthesis, yield, and quality [41, 42]. It is well established that nitrogen, carbon, and water cycles are closely interrelated during plant growth [43, 44]. Nitrogen metabolism depends on carbon metabolism for carbon skeletons and energy, while carbon metabolism relies on nitrogen metabolism for enzyme production and photosynthetic pigment synthesis [44].

Previous results showed that the relative transcription level of *NtANTL2* was significantly higher in mild aromatic tobacco varieties Weining and Tianzhu than in medium aromatic Kaiyang, suggesting that *NtANTL2* may be associated with amino acid content and transport. However, the mechanism by which the ANT2 family participates in the uptake and long-distance transport of nitrogenous organic compounds remains unclear. This study reports the characterization of *NtANTL2* gene function in tobacco growth, enzyme activity, carbon and nitrogen metabolism, and amino acid accumulation under varying nitrogen applications.

## Material and methods

### Plant materials and culture condition

Tobacco variety K326 was kindly provided by China National Tobacco Corporation. Plants were cultivated in 500 mL pots containing a soil mixture (50% vermiculite) at 25 °C under a 16 h light/8 h dark photoperiod, with a light intensity of 52 μmol·m⁻^2^·s⁻^1^ and relative humidity of 80%. Urea was added to achieve final nitrogen concentrations of 0.1%, 0.2%, and 0.3%.

### *NtANTL2* transgenic plant vector construction and genetic transformation

The pCAMBIA2301G vector, *Agrobacterium tumefaciens* strain LBA4404, and PEASY-Blunt vector were provided by the College of Life Science, Yangtze University. The pCAMBIA2301G vector contains the CaMV 35S promoter, which is a constitutive promoter known for driving gene expression in tobacco. RNA was extracted from tobacco K326 plants and reverse-transcribed to synthesize complementary DNA (cDNA). The *NtANTL2* gene was amplified using a high—fidelity Pfu DNA polymerase, and the resulting fragment was ligated downstream of the CaMV 35S promoter in the pCAMBIA2301G expression vector to ensure its expression in transgenic plants. *Escherichia coli* DH5α was transformed with the recombinant pCAMBIA2301G-NtANTL2 plasmid. Positive clones were identified, plasmid DNA was extracted, and transformed into *Agrobacterium tumefaciens* LBA4404. Selected colonies were cultured and stored at − 80 °C. Tobacco was transformed using the *Agrobacterium*-mediated method with K326 leaf discs as explants to generate T3 *NtANTL2*-overexpressing transgenic plants. RT-qPCR analysis confirmed the overexpression of *NtANTL2* in transgenic lines. After 30 days of growth, both control and transgenic tobacco plants were harvested for further analysis. All the primers used were listed in Supplemental Table S1.

### RNA extraction and qPCR analysis

*OE-ANTL2-3* and WT tobacco plants were cultivated to the six-leaf stage, and plants with similar growth were transplanted to soil with nitrogen levels of 0.1%, 0.2%, and 0.3%. Total RNA was extracted from approximately 100 mg of tobacco leaves using TRIzol reagent, following the manufacturer’s instructions (Invitrogen, USA). One microgram of total RNA was reverse-transcribed using the PrimeScript™ II 1 st Strand cDNA Synthesis Kit (TaKaRa, Japan). The diluted cDNA (fivefold) was used as the template for RT-qPCR analysis using a CFX Connect Real-Time System (BIO-RAD). Relative transcription levels of *NtANTL2-3* genes, carbon metabolism-related genes (*GBSSI*, *SUS*, *INV*, *SPS*), and nitrogen metabolism-related genes (*GDH*, *NR*, *GS*, *GOGAT*) were analyzed. Relative expression levels were calculated using the 2⁻ΔΔCT method [45]. All the primers used for RT-qPCR were listed in Supplemental Table S1. All experiments were repeated at least three times with similar results.

### Morphological and physiological measurements of tobacco seedlings

Uniformly grown seedlings at the six-leaf stage were planted in pots with varying nitrogen concentrations. Plant height and root length were measured after 30 days, and fresh/dry weights of stems, leaves, and roots were recorded. Chlorophyll content were analyzed by spectrophotometer method [46]. Soluble carbohydrate and protein contents were measured following established protocols [47, 48]. Enzyme activities, including sucrose invertase, sucrose synthase, sucrose phosphate synthase, nitrate reductase, glutamine synthetase, and glutamate synthase, were measured as described previously [15, 16, 49, 50]. Four biological replicates with five plants per replicate were used.

### Nitrogen treatment and phenotypic analysis

Full and pest-free K326 (Wester, WT) seeds and T3 generation of overexpressing *NtANTL2* transgenic (OE-ANTL2) seeds were disinfected in 75% alcohol for 30 s and then washed with sterile water for 2–3 times. After disinfection, K326 seeds were inoculated on MS medium, and OE-ANTL2 seeds were inoculated on 100 mg L^−1^ kanamycin + MS medium. Plants were cultivated under dark conditions 2 days, and then transferred to conditions with a photoperiod of 16 h light/8 h darkness at 25 ℃ for about 30 days.

Soil samples were collected from the field, air-dried, and mixed with a matrix at a 1:1 volume ratio. Initial soil N content was 0.097%, measured via the Kjeldahl method: samples were digested with concentrated sulfuric acid and a catalyst to convert N into ammonium, which was then alkalized, distilled, and absorbed by boric acid before titration with standard acid to calculate total N. Urea was added to adjust mixed substrate N levels to 0.1% (control), 0.15%, 0.2%, 0.3%, 0.4%, and 0.5%. After transplanting tobacco seedlings into these substrates, high-N treatments (0.4% and 0.5%) caused chlorosis or plant death within 20 days, while plants under 0.1%, and 0.15% N application showed no significant differences. Thus, 0.1%, 0.2%, and 0.3% N concentrations were selected for further experiments. Four biological replicates were performed, each containing five plants.

### Amino acids analysis

Metabolomic analysis of amino acids was performed by Wuhan Metware Biotechnology Co., Ltd. Samples were lyophilized, crushed into powder, and extracted with 70% aqueous methanol. The extracts were analyzed using an ultra-performance liquid chromatography–tandem mass spectrometry (UPLC–MS/MS) system. Amino acids with a variable importance in projection ≥ 1 and an absolute log_2_ fold change (log_2_FC) ≥ 1 were considered differentially regulated. Three biological replicates were conducted, with four plants included in each replicate.

### Statistical analysis

One-way two-factor ANOVA followed by Fisher’s protected LSD multiple comparison was used to evaluate differences among conditions. Statistical analyses were performed using SPSS 20.0 (Chicago, USA), and significance was indicated by different letters (a, b, c) for *p* < 0.05.

## Results

### *NtANTL2* overexpression enhances growth under high nitrogen conditions

The *NtANTL2* gene family in tobacco comprises five copies (*NtANTL2-1* to *NtANTL2-5*), with tissue-specific expression patterns suggesting functional specialization across tissues. Quantitative analysis revealed that *NtANTL2-1*, *NtANTL2-2*, *NtANTL2-4*, and *NtANTL2-5* exhibited the highest transcript levels in leaves, followed by stems, with minimal expression in roots. In contrast, *NtANTL2-3* displayed preferential expression in stems (Figure S1). Five stable overexpression lines (*OE-ANTL2-1* to *OE-ANTL2-5*) were generated, and OE-ANTL2-3 exhibited the highest *NtANTL2* expression among transgenic lines and it was selected for detailed phenotypic and molecular characterization. Relative expression analysis of *NtANTL2-3* in transgenic and wild-type (WT) plants under varying nitrogen regimes is presented in Figure S2.

**Fig. 1:**
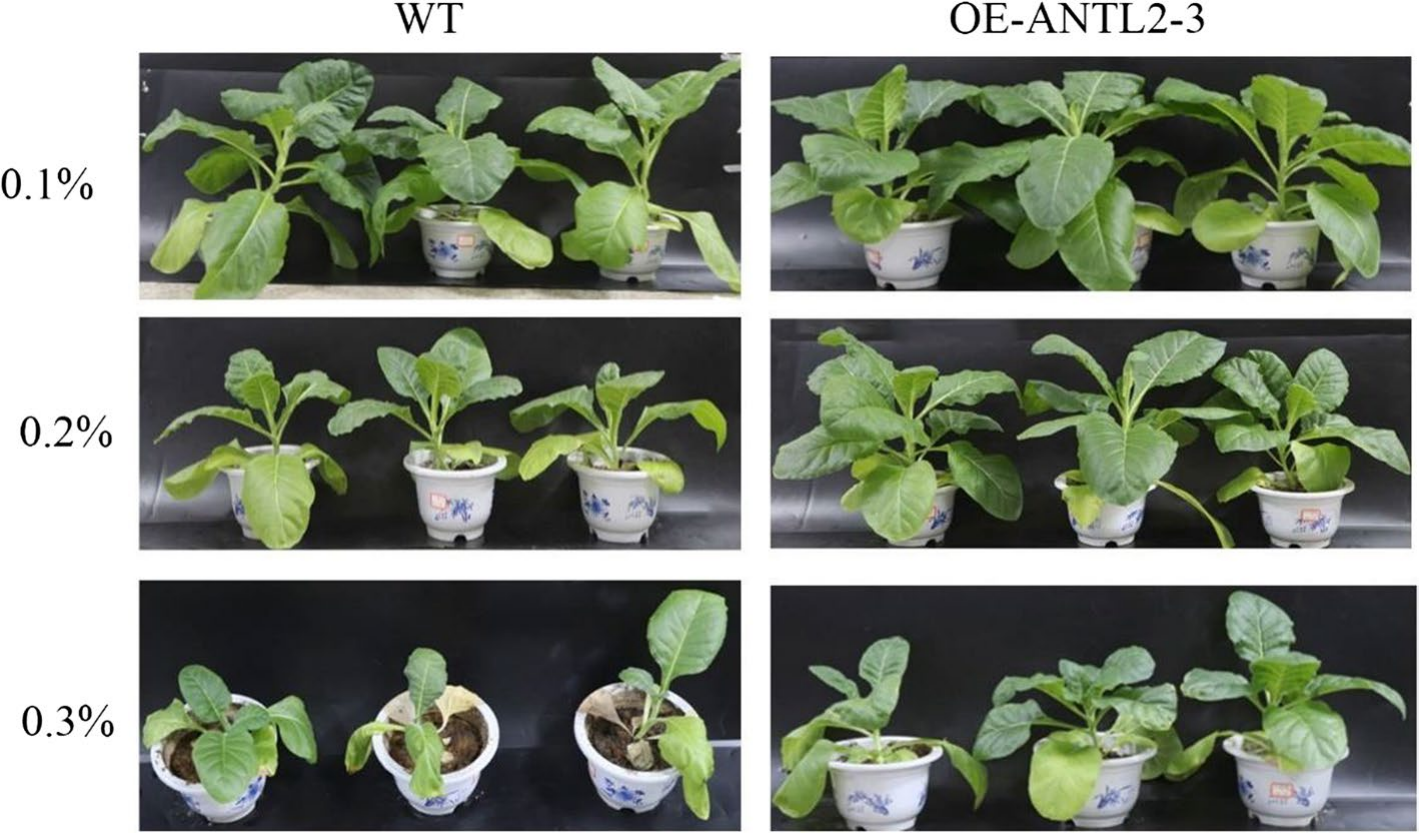
Growth of wide type and OE-ANTL2-3 plants under different nitrogen conditions

Tobacco seedlings (OE-ANTL2-3 and WT) were cultivated to the six-leaf stage before transplantation into substrates amended with 0.1%, 0.2%, or 0.3% nitrogen. After 30 days of treatment, OE-ANTL2-3 plants exhibited significantly enhanced agronomic traits compared to WT plants, including increased leaf length, leaf width, and plant height (Fig. 1), indicating improved growth performance under tested N conditions.

At a nitrogen level of 0.1%, WT plants exhibited greater plant height than *OE*-*ANTL2-3* plants, but other traits showed no significant differences. At 0.2% nitrogen, *OE-ANTL2-3* plants demonstrated significantly higher plant height, shoot fresh weight, and root length than WT plants. At 0.3% nitrogen, *OE*-*ANTL2-3* plants showed remarkable increases in plant height (2.31 times), shoot fresh weight (3.23 times), root length (1.47 times), root fresh weight (6.06 times), and root dry weight (5.67 times) compared to WT plants. These results suggest that *OE*-*ANTL2-3* plants have enhanced tolerance to high nitrogen levels (Table 1).

**Table 1:** Investigation on agronomic characters of transgenic plants and wild type plants under different nitrogen levels

Nitrogen %	Material	Plant height (cm)	Shoot FW (g)	Shoot DW (g)	Root length (cm)	Root FW (g)	Root DW (g)
0.1	WT	63.33 ± 1.20a	165.67 ± 6.39a	14.81 ± 1.24a	11.27 ± 0.15a	7.00 ± 0.58a	1.18 ± 0.11a
	OE-ANTL2-3	49.33 ± 1.76b	148.33 ± 10.53ab	12.85 ± 1.66ab	10.00 ± 0.58ab	6.50 ± 0.29a	0.79 ± 0.21ab
0.2	WT	33.33 ± 1.76c	113.00 + 3.00b	6.03 ± 0.32c	7.67 ± 0.17c	2.00 ± 0.00 cd	0.30 ± 0.02 cd
	OE-ANTL2-3	46.33 ± 1.86b	163.33 ± 16.76a	10.15 ± 1.41b	9.33 ± 0.44b	3.33 ± 0.67bc	0.70 ± 0.19bc
0.3	WT	19.33 ± 3.18 d	44.00 ± 21.39c	2.39 ± 1.26c	6.33 ± 0.33c	0.77 ± 0.15 d	0.12 ± 0.03 d
	OE-ANTL2-3	44.67 ± 3.93b	142 ± 9.64ab	10.17 ± 1.41b	9.33 ± 0.67b	4.67 ± 0.88b	0.68 ± 0.11bc

The data in the table are mean ± standard error. Different lowercase letters in the same column indicated a difference of 0.05 levels

### Effects of *NtANTL2* overexpression on carbon metabolism

**Fig. 2:**
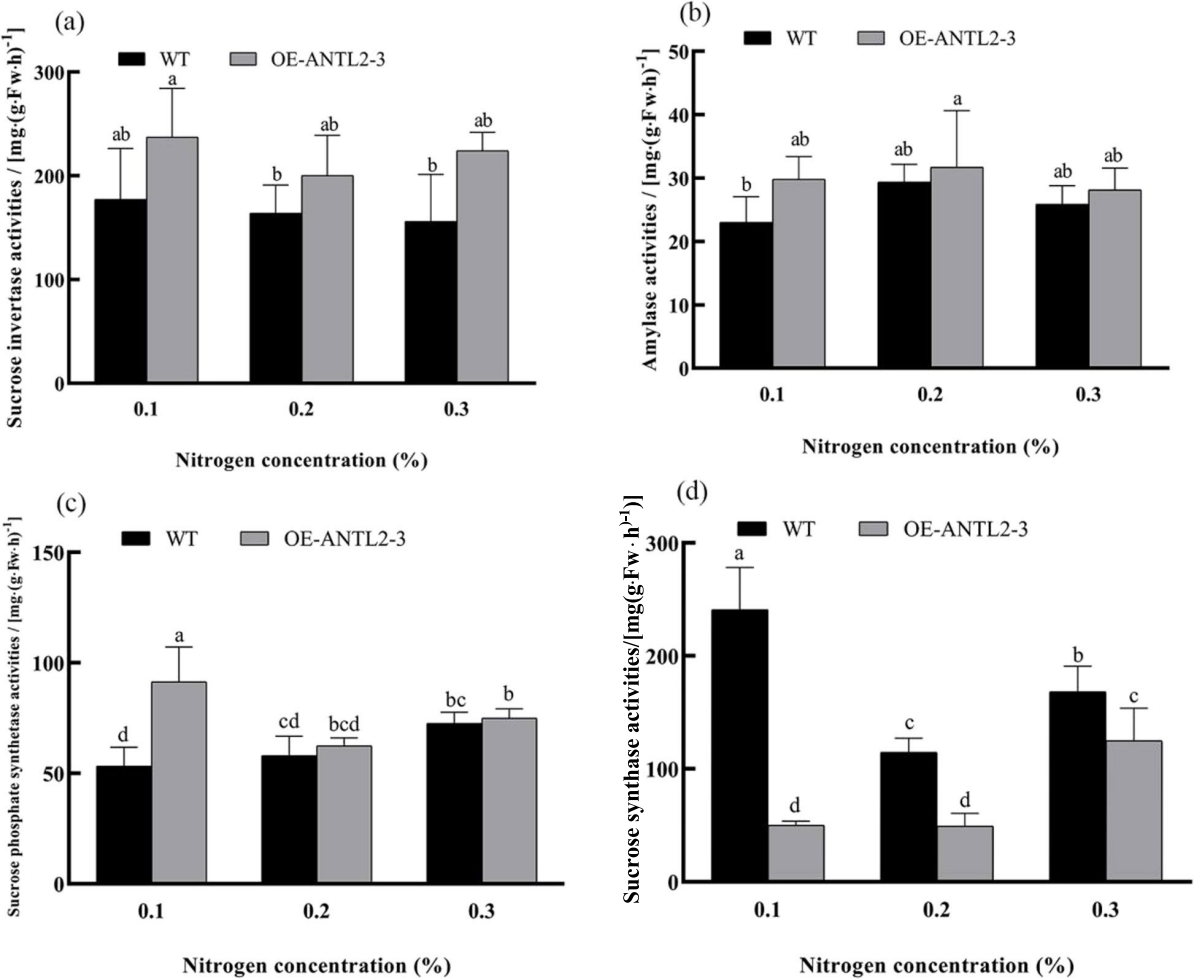
Comparison of carbon metabolism related enzyme activities between transgenic plants and wild plants under different nitrogen levels

Carbon metabolism is a fundamental process in tobacco leaves, and its regulation is closely linked to the activity of associated enzymes. Enzyme activities of sucrose invertase (EC 3.2.1.26), α-amylase (EC 3.2.1.1), sucrose phosphate synthetase (2.4.1.14), and sucrose synthase (EC 2.4.1.13) were measured in WT and *OE-ANTL2-3* plants under varying nitrogen levels. Sucrose invertase and amylase activities in *OE-ANTL2-3* plants were similar to those in WT plants across all three nitrogen levels (Fig. 2a and b). Sucrose phosphate synthetase activity in *OE-ANTL2-3* plants was 1.5 times higher than in WT plants at 0.1% nitrogen but showed no significant differences at 0.2% and 0.3% nitrogen levels (Fig. 2c). Conversely, sucrose synthase activity in *OE-ANTL2-3* plants was significantly lower than in WT plants under all nitrogen levels, decreasing by 79.23% and 57.03% at 0.1% and 0.2%, respectively. At 0.3% nitrogen, *OE-ANTL2-3* plants exhibited higher sucrose synthase activity than at lower nitrogen levels (Fig. 2d).

**Fig. 3:**
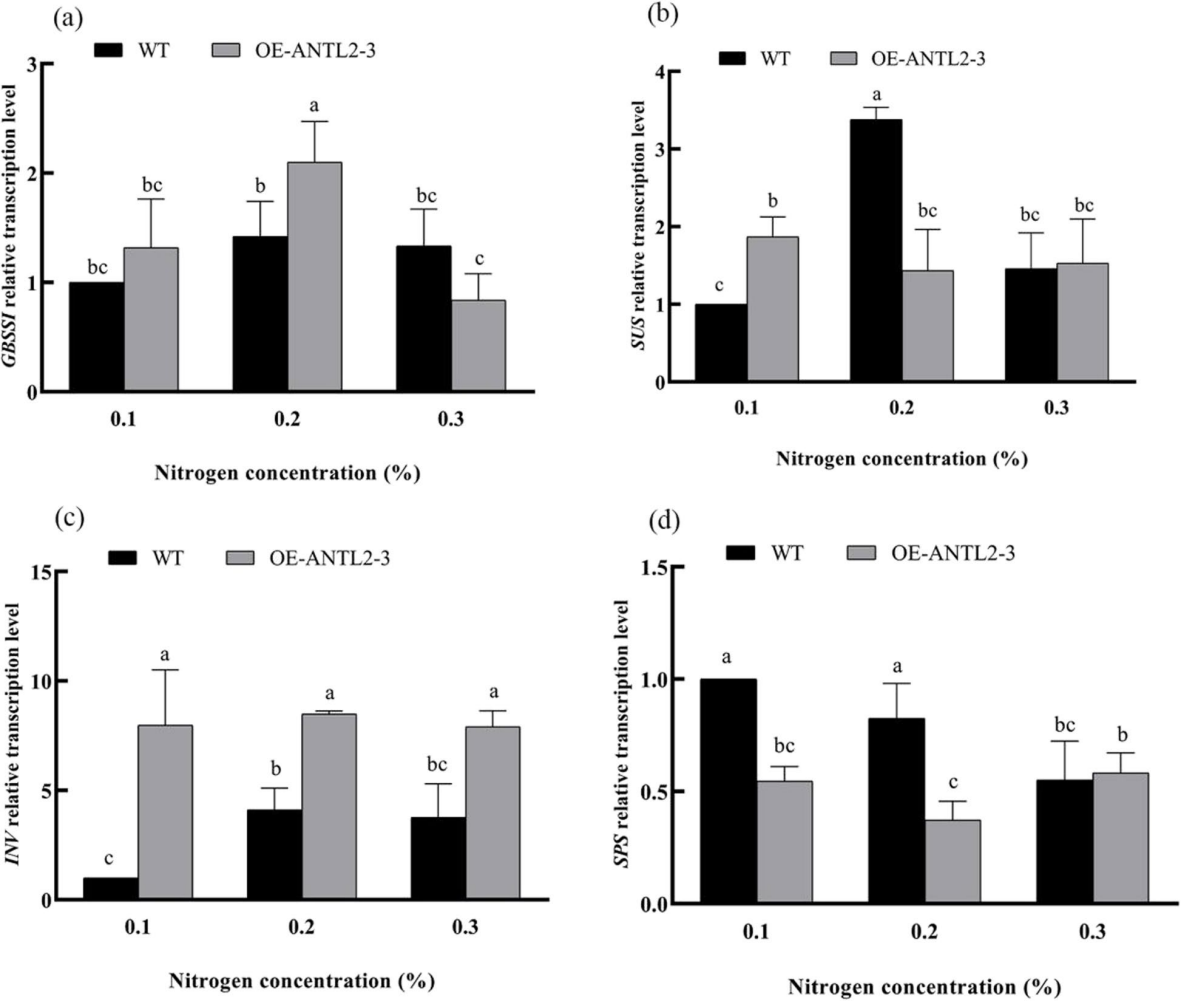
Comparison of transcription levels of carbon metabolism related genes between transgenic plants and wild plants under different nitrogen levels

Expression levels of carbon metabolism-related genes were also analyzed under different nitrogen levels. In *OE-ANTL2-3* plants, the relative transcription levels of *GBSSI*, *SUS*, *INV*, and *SPS* genes varied significantly. For example, *GBSSI* expression was 47.82% higher at 0.2% nitrogen, and *INV* expression was consistently higher than WT across all nitrogen levels (Fig. 3). Regarding the discrepancies between gene expression and enzyme activity, potential post—transcriptional and post—translational regulation mechanisms could cause the differences.

### Effects of *NtANTL2* overexpression on nitrogen metabolism

Key nitrogen metabolism enzymes, including nitrate reductase (NR, EC 1.6.6.1), glutamine synthetase (GS, EC 6.3.1.2), glutamate synthase (GOGAT, EC 1.4.1.14), and glutamate dehydrogenase (GDH, EC 1.4.1.2), were analyzed. GDH activity in *OE-ANTL2-3* plants was significantly higher than in WT plants at 0.1% and 0.3% nitrogen, increasing by 236.84% and 425.00%, respectively. The relative transcription level of *GDH* was also elevated in *OE-ANTL2-3* plants at lower nitrogen levels.

Glutamine (Gln) is the first amino acid synthesized in the nitrogen assimilation process in plants. GS catalyzes the conversion of glutamate (Glu) and NH4^+^ into glutamine at the expense of ATP. Tobacco plants primarily absorb nitrate (NO3^−^) and ammonium (NH4^+^) from the soil through their roots. Nitrate must be reduced by NR and nitrite reductase (NiR, EC 1.7.7.1) to ammonia, which is then assimilated into amino acids through the GS and GOGAT system, a crucial step in nitrogen metabolism.

**Fig. 4:**
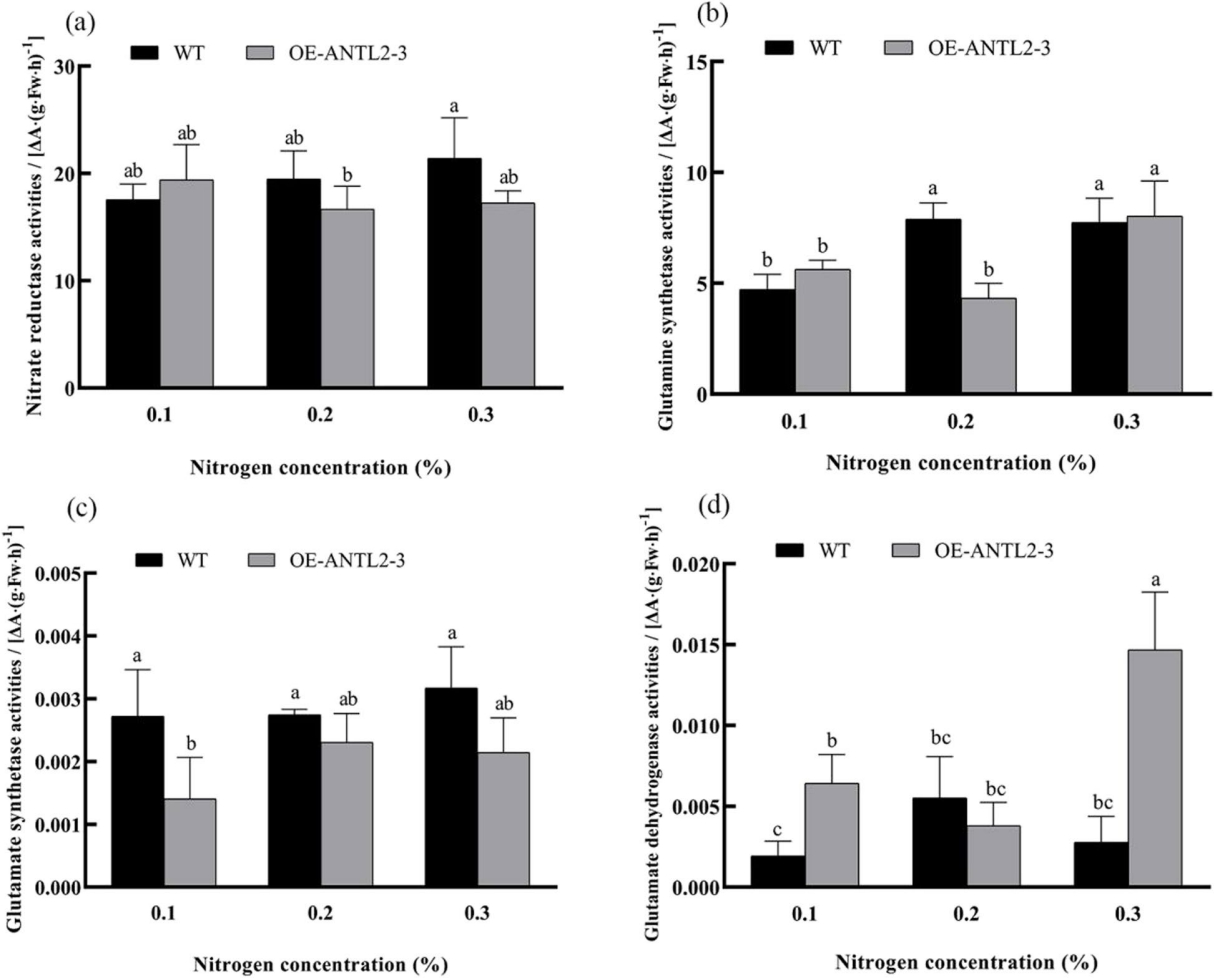
Comparison of nitrogen metabolism related enzyme activities between transgenic plants and wild plants under different nitrogen levels

The NR activity in *OE-ANTL2-3* and WT plants is comparable (Fig. 4). The GS activity in *OE*-*ANTL2-3* plants is lower than that in WT plants at a 0.2% nitrogen level, with a decrease of 45.20%. At other nitrogen levels, no difference is found between *OE*-*ANTL2-3* plants and WT plants. The GOGAT activity of *OE*-*ANTL2-3* plants is lower than that of WT plants at a 0.1% nitrogen level, showing a reduction of 48.15%. The GDH activity of *OE-ANTL2-3* plants at 0.1% and 0.3% nitrogen levels is significantly higher than that of WT plants, increasing by 236.84% and 425.00% respectively. The GDH activity of WT plants remains unchanged among all nitrogen levels. The GDH activity of *OE-ANTL2-3* plants at a 0.3% nitrogen level is significantly higher than that at 0.1% and 0.2% nitrogen levels, with increases of 129.69% and 286.84%, respectively.

**Fig. 5:**
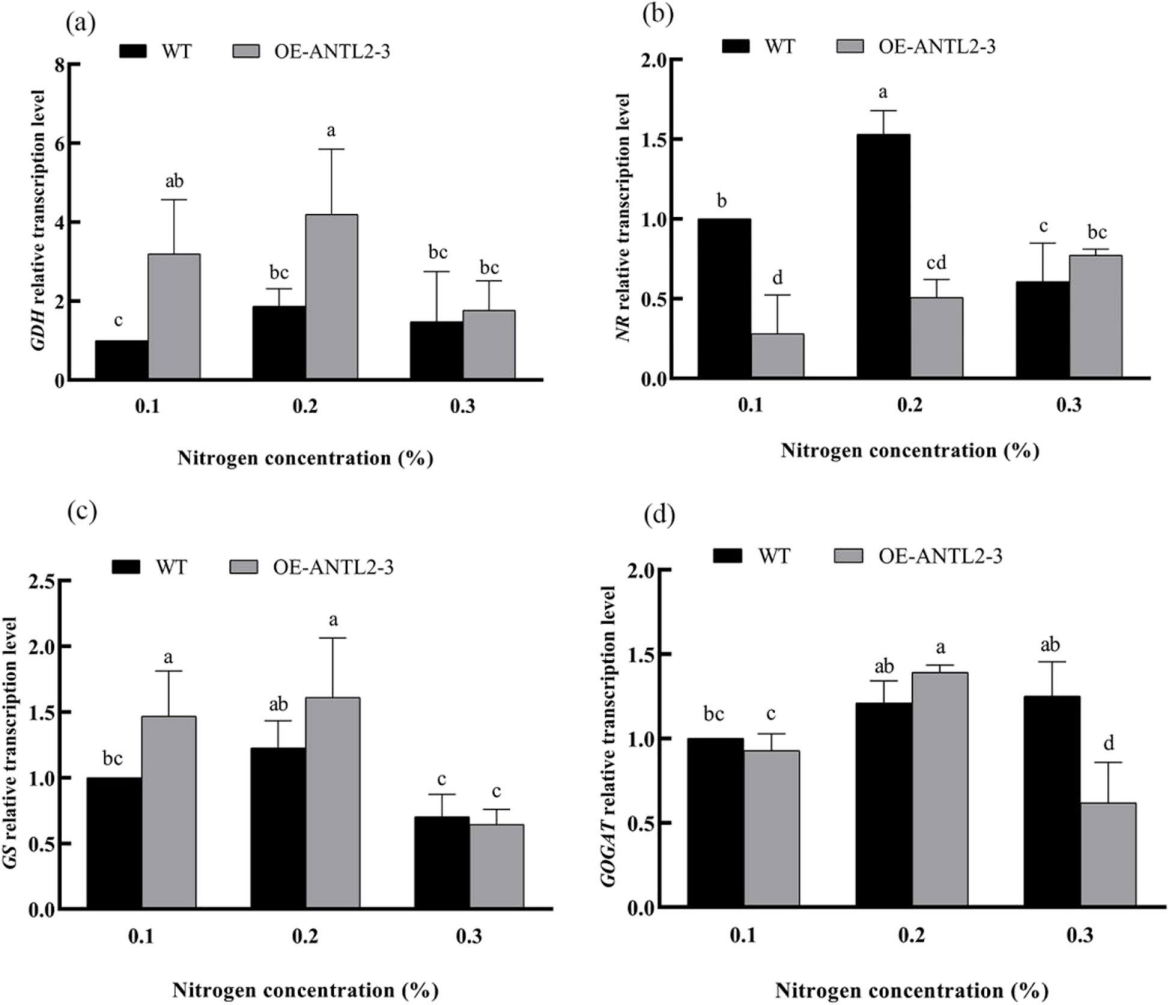
Comparison of transcription levels of nitrogen metabolism related genes between transgenic plants and wild plants under different nitrogen levels

The gene expression levels of *GDH*, *NR*, *GS*, and *GOGAT* were analyzed under three different nitrogen levels. The relative transcription level of the *GDH* gene in *OE-ANTL2-3* plants is higher than that in WT plants at 0.1% and 0.2% nitrogen levels, with increases of 219.22% and 124.07% respectively. At 0.1% and 0.2% nitrogen levels, the relative transcription level of the *NR* gene is significantly lower than that of WT plants, decreasing by 72.02% and 66.91% respectively. Under a 0.1% nitrogen level, the relative transcription level of the GS gene is significantly higher than that of WT plants, increasing by 46.66%. Under 0.3% nitrogen level, the relative transcription level of the *GOGAT* gene is significantly lower than that of WT plants, decreasing by 46.21%. The enzyme activity and gene expression of *GS* and *GDH* were consistent. In OE plants, GS activity was lower than wide type plants (Figs. 4 and 5).

**Fig. 6:**
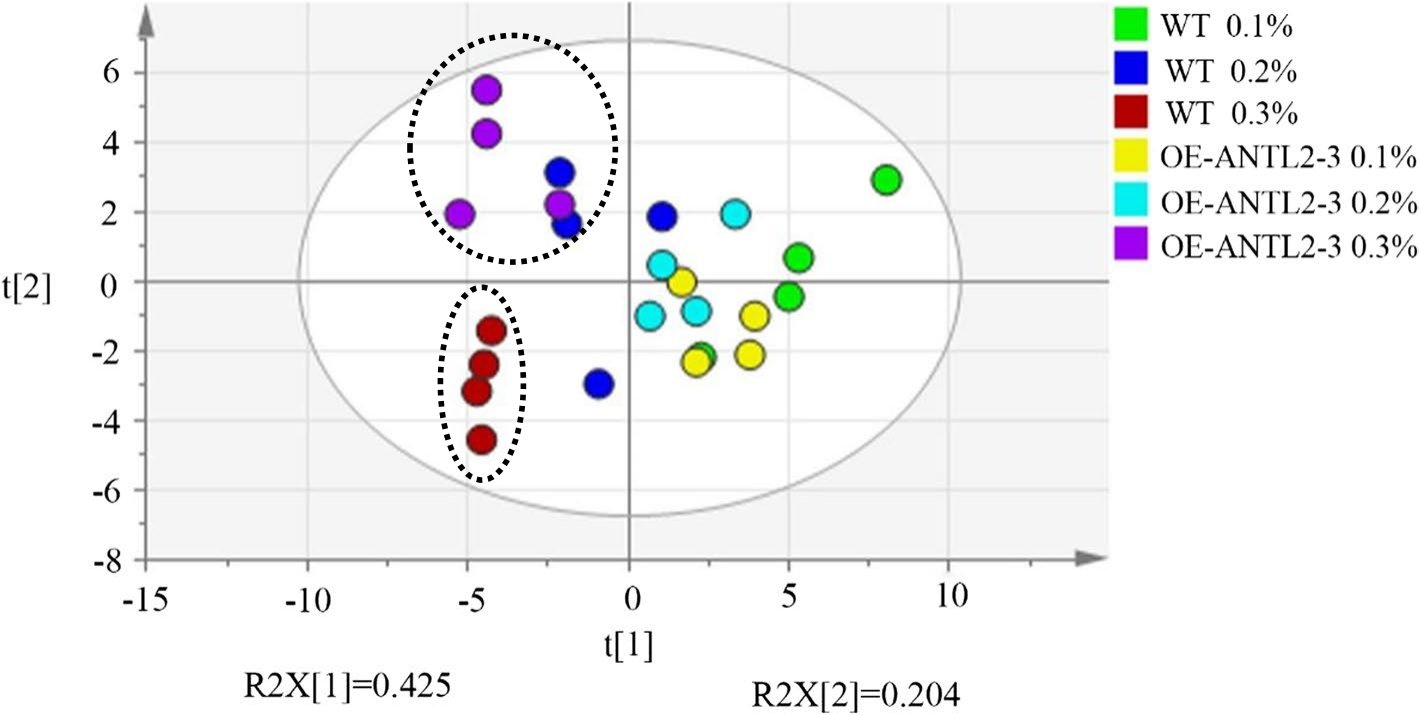
Scatter plots (PCA plots) of principal component analysis of nitrogen containing substances in transgenic plants and wild plants under different nitrogen levels. Three biological replicates were conducted, with four plants included in each replicate. Unit: ug/g dry weight

According to the principal component analysis of nitrogen compounds content in *OE-ANTL2-3* plants and WT plants (Fig. 6), the contents of nitrogen substances in tobacco leaves of *OE-ANTL2-3* plants and WT plants were different under different nitrogen levels, among which the contents of nitrogen substances in tobacco leaves of *OE-ANTL2-3* plants and WT plants were the most different under 0.3% nitrogen level. They were obviously divided into two categories. Therefore, the subsequent analysis focused on the difference of nitrogen content in tobacco leaves between *OE-ANTL2-3* plants and WT plants at 0.3% nitrogen level.

### Effects of *NtANTL2* Overexpression on amino acid metabolism

As can be seen from Table 2, the metabolites with significant differences (*P* < 0.05) in nitrogen content of tobacco leaves between *OE-ANTL2-3* plants and WT plants include: aspartic acid, glycine, threonine, arginine, gamma-aminobutyric acid, tyrosine, valine, histidine, methionine, leucine, phenylalanine, ornithine, lysine, tyramine, putrescine, phenylethylamine, and isoamylamine. At 0.3% nitrogen level, the content of aspartic acid in *OE-ANTL2-3* plants was significantly lower than that in WT plants, and the contents of glycine, threonine, and tyrosine were significantly increased, while the contents of histidine, arginine, gamma-aminobutyric acid, valine, methionine, leucine, phenylalanine, ornithine, lysine, tyramine, putputylamine, phenethylamine and isoamylamine were significantly increased.

**Table 2:** Multiple comparisons of nitrogenous substances in tobacco leaves of transgenic plants and wild plants under 0.3% nitrogen level (unit: ug/g dry weight)

Parameters	WT 0.3%	OE-ANTL2-3 0.3%	Sig
aspartic acid	1261.6 ± 223.91	816.02 ± 92.83	0.010
histidine	174.04 ± 15.65	306.4 ± 79.62	0.020
glycine	50.78 ± 4.35	75.03 ± 5.53	0.004
threonine	418.68 ± 34.8	519.76 ± 27.26	0.009
arginine	175.89 ± 26.98	343.33 ± 82.26	0.011
γ-aminobutyric acid	212.9 ± 27.3	270.94 ± 28.14	0.025
alanine	373.37 ± 32.77	389.09 ± 30.05	0.506
proline	247.45 ± 46.65	329.6 ± 46.41	0.069
tyrosine	111.56 ± 23.6	221.07 ± 41.29	0.006
valine	180.29 ± 14.73	267.92 ± 48.27	0.017
methionine	34.64 ± 4.6	41.94 ± 1.57	0.049
tryptophan	129.49 ± 19.06	208.66 ± 61.54	0.055
leucine	193.22 ± 13.43	286.57 ± 62.61	0.031
phenylalanine	172.54 ± 13.59	239.39 ± 38.52	0.022
ornithine	3.50 ± 0.83	5.8 ± 0.73	0.013
lysine	147.49 ± 27.78	261.9 ± 48.01	0.010
tyramine	124.71 ± 33.69	214.32 ± 34.71	0.018
tetrametnylened iamine	149.26 ± 33.31	310.92 ± 83.51	0.011
phenylethylamine	45.36 ± 10.34	70.54 ± 15.02	0.033
isoamylamine	584.15 ± 62.71	1227.7 ± 260.86	0.014

Meanwhile, the chlorophyll, soluble sugar, soluble protein, total nitrogen, and total protein contents in transgenic and wild plants under different nitrogen levels were analyzed. Results showed no differences in these parameters between the two plant types, indicating genetic modification did not affect their physiological substance levels in response to varying nitrogen conditions (Fig. S3, S4, and S5).

## Discussion

Nitrogen, a critical macronutrient for plant growth, often constrains crop yield due to its limited availability and the intricate balance required for optimal carbon -nitrogen metabolism. Efficient N use hinges on dynamic partitioning between source (e.g., leaves) and sink (e.g., roots, reproductive organs) tissues, which integrates N uptake, photosynthetic enzyme activity (e.g., Rubisco, phosphoenolpyruvate carboxylase), and phloem-mediated amino acid transport [51, 52]. Disruptions in this balance—such as excessive N fertilization—can lead to soil salinization, nitrate accumulation, and stunted growth, underscoring the need to develop crops with improved N tolerance and utilization [3, 53].

In this study, overexpression of *NtANTL2*, a tobacco amino acid transporter gene, demonstrated enhanced tolerance to high N conditions, primarily through coordinated regulation of C-N metabolism. Transgenic plants exhibited superior agronomic traits, including increased biomass and leaf dimensions, even at elevated N levels (Table 1, Fig. 1), aligning with the pivotal role of amino acid transporters in mediating source-sink nutrient allocation [54, 55]. Maintaining stable chlorophyll content under high N stress suggests preserved photosynthetic capacity, a critical factor for sustaining C assimilation and NUE [56, 57].

Nitrate reductase (NR), a key enzyme in N assimilation, showed increased activity in *NtANTL2*-overexpressing plants, indicating enhanced conversion of nitrate to nitrite and subsequent amino acid biosynthesis [58, 59]. This was corroborated by elevated levels of specific amino acids (e.g., histidine, arginine, γ-aminobutyric acid), which serve as both N storage molecules and C skeletons for energy metabolism [60, 61]. Conversely, reduced aspartic acid content in transgenic lines may reflect reallocation of C skeletons toward more N-efficient metabolic pathways, illustrating the role of *NtANTL2* in fine-tuning C-N balance [62, 63].

The tissue-specific expression of *NtANTL2* family members—with most copies preferentially expressed in leaves and stems, and *NtANTL2-3* enriched in stems—suggests functional specialization in organ-specific N transport (Fig. S1). This aligns with previous findings where amino acid transporters participate in phloem loading/unloading and long-distance N translocation, with mutants like *ant1* showing disrupted sieve tube amino acid dynamics [11, 38]. By enhancing amino acid allocation to sinks and optimizing N metabolism, *NtANTL2* overexpression likely mitigates the negative effects of high N, such as reduced photosynthesis and metabolic imbalance [28, 31].

Amino acid transporters are central to integrating environmental cues (e.g., nitrate availability) with metabolic responses, as evidenced by nitrate-inducible expression of homologous *ANT* genes in *Arabidopsis* and wheat [37]. Here, *NtANTL2*’s role in upregulating N assimilation genes and improving N-containing compound levels under high N stress highlights its potential as a molecular target for breeding nitrogen-efficient tobacco cultivars. Such strategies are vital for sustainable agriculture, addressing both yield stability and environmental concerns associated with excessive fertilizer use [64, 65].

While this study establishes *NtANTL2* as a key regulator of N tolerance and C-N metabolism, unresolved questions remain about the precise signaling pathways and interacting proteins mediating its effects. Elucidating these mechanisms will deepen our understanding of amino acid transporter multifunctionality—including roles in stress resistance and pathogen defense—and enable targeted manipulation of metabolic networks to enhance crop productivity under challenging N regimes [33, 66].

In conclusion, *NtANTL2* overexpression improves high N tolerance in tobacco by optimizing amino acid transport, enhancing N assimilation, and balancing C-N metabolism. These findings provide a foundation for developing molecular breeding strategies that reconcile agricultural productivity with environmental sustainability, underscoring the importance of amino acid transporters in shaping crop responses to nutrient stress.

## Conclusion

Overexpression of *NtANTL2* significantly improves plant nitrogen uptake and utilization under high nitrogen conditions. Under normal nitrogen conditions, WT plants exhibit better growth than *NtANTL2*-overexpressing plants; however, *NtANTL2*-overexpressing plants demonstrate superior tolerance to high nitrogen stress. These plants show increased levels of histidine, arginine, gamma-aminobutyric acid, valine, methionine, leucine, phenylalanine, tryptophan, ornithine, lysine, tyramine, putrescine, phenylethylamine, and isovaleric acid, while aspartic acid levels are reduced. Based on previous findings that *NtANTL2* inhibition significantly increases aspartic acid content (data not shown), it can be concluded that *NtANTL2* regulates nitrogen uptake and influences amino acid composition in tobacco, ultimately affecting the plant's carbon and nitrogen metabolism. These findings provide a molecular basis for breeding high-efficiency nitrogen use tobacco varieties.

## Supplementary Information

Supplementary Material 1: Fig. S1 Identification of the relative transcription levels of NtANTL2-3 in transgenic plants and wild-type plants. Fig. S2 Identification of the relative transcription levels of NtANTL2-3 in transgenic plants and wild type plant. Fig. S3 Comparison of chlorophyll contents between transgenic plants and wild plants under different nitrogen levels. Fig. S4 Comparison of soluble sugar and soluble protein contents between transgenic plants and wild plants under different nitrogen levels. Fig. S5 Comparison of total nitrogen contents and total protein contents between transgenic plants and wild plants under different nitrogen levels.

Supplementary Material 2: Supplementary Table 1. Primer names and sequences of clone NtANTL2.

## Data Availability

All raw data and plant material can be provided by authors.

## Abbreviations

NUE: Nitrogen use efficiency
ACC: 1-aminocyclopropane-1-carboxylate
NR: Nitrate reductase
GS: Glutamine synthetase
GOGAT: Glutamate synthase
GDH: Glutamate dehydrogenase
NR: Nitrate reductase
NiR: Nitrite reductase
LHT1: Lysine histidine transporter 1
AAP: Amino acid/auxin permeases
WT: Wild-type
GBSSI: Granule - bound starch synthase I
SUS: Sucrose synthase
INV: Invertase
SPS: Sucrose phosphate synthase
Gln: Glutamine
Gln: Glutamate
