# Supplementary material for: NtANTL2 overexpression regulates starch-related and nitrogen metabolism in tobacco plants
Source: BMC Plant Biol. 2025 Jun 7;25:775. doi: 10.1186/s12870-025-06748-8 (PMC12144802; doi:10.1186/s12870-025-06748-8)
Supplement: Supplementary file 1 — Supplementary Material 1: Fig. S1 Identification of the relative transcription levels of NtANTL2-3 in transgenic plants and wild-type plants. Fig. S2 Identification of the relative transcription levels of NtANTL2-3 in transgenic plants and wild type plant. Fig. S3 Comparison of chlorophyll contents between transgenic plants and wild plants under different nitrogen levels. Fig. S4 Comparison of soluble sugar and soluble protein contents between transgenic plants and wild plants under different nitrogen levels. Fig. S5 Comparison of total nitrogen contents and total protein contents between transgenic plants and wild plants under different nitrogen levels. [file 12870_2025_6748_MOESM1_ESM.pdf]

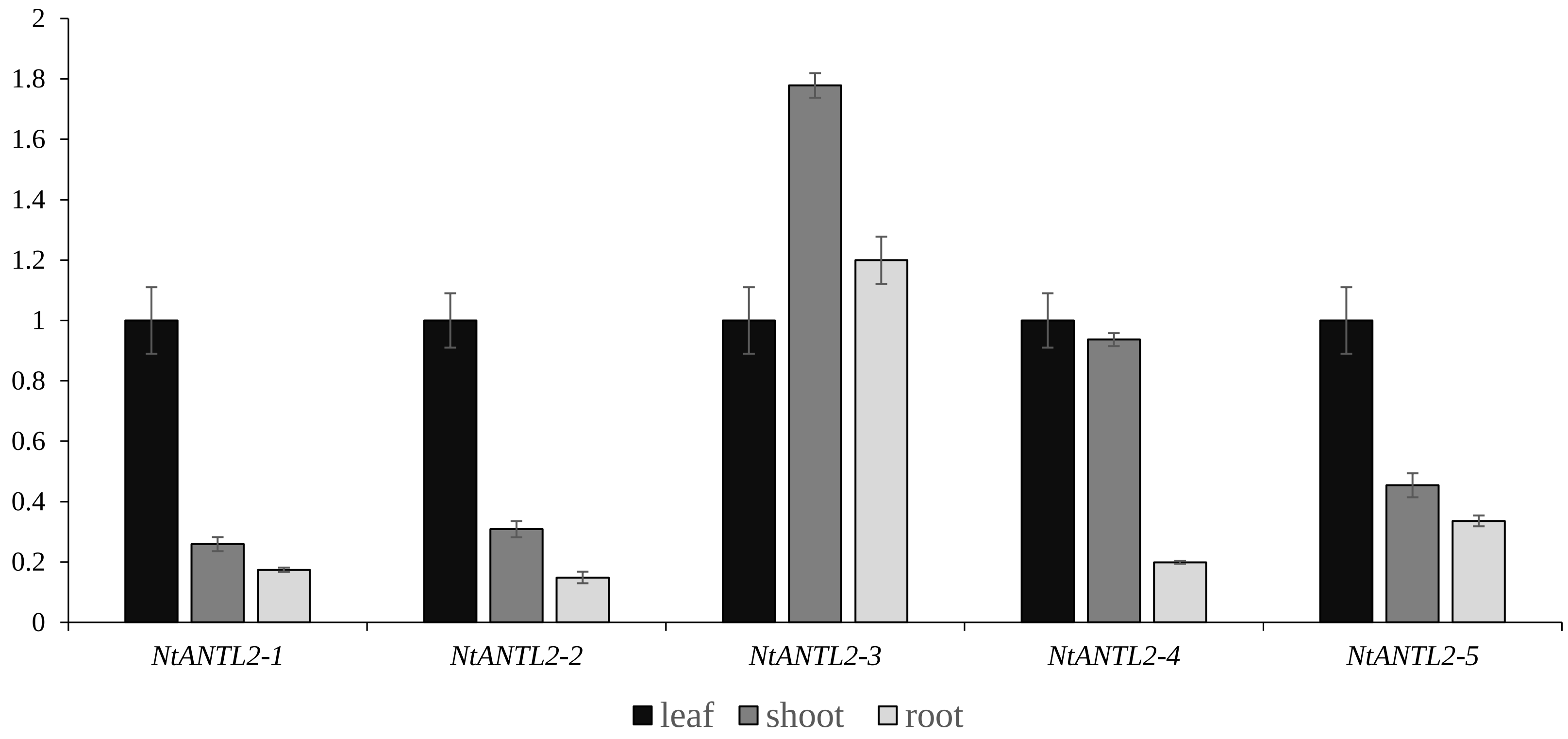

Fig.S1 The expression levels of the NtANTL2 gene in different parts of tobacco K326 at different periods

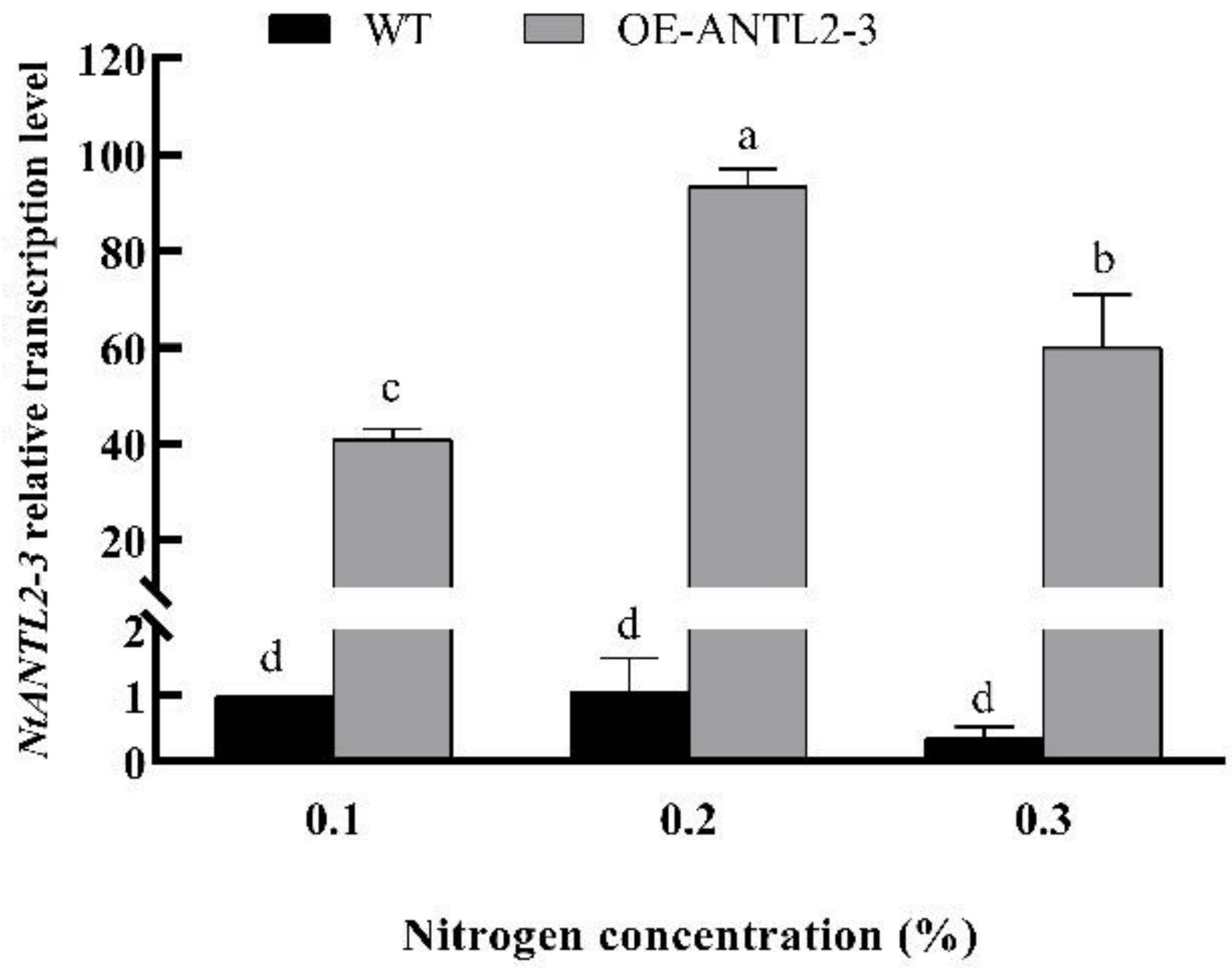

Fig.S2 Identification of the relative transcription levels of *NtANTL2*-3 in transgenic plants and wild-type plants

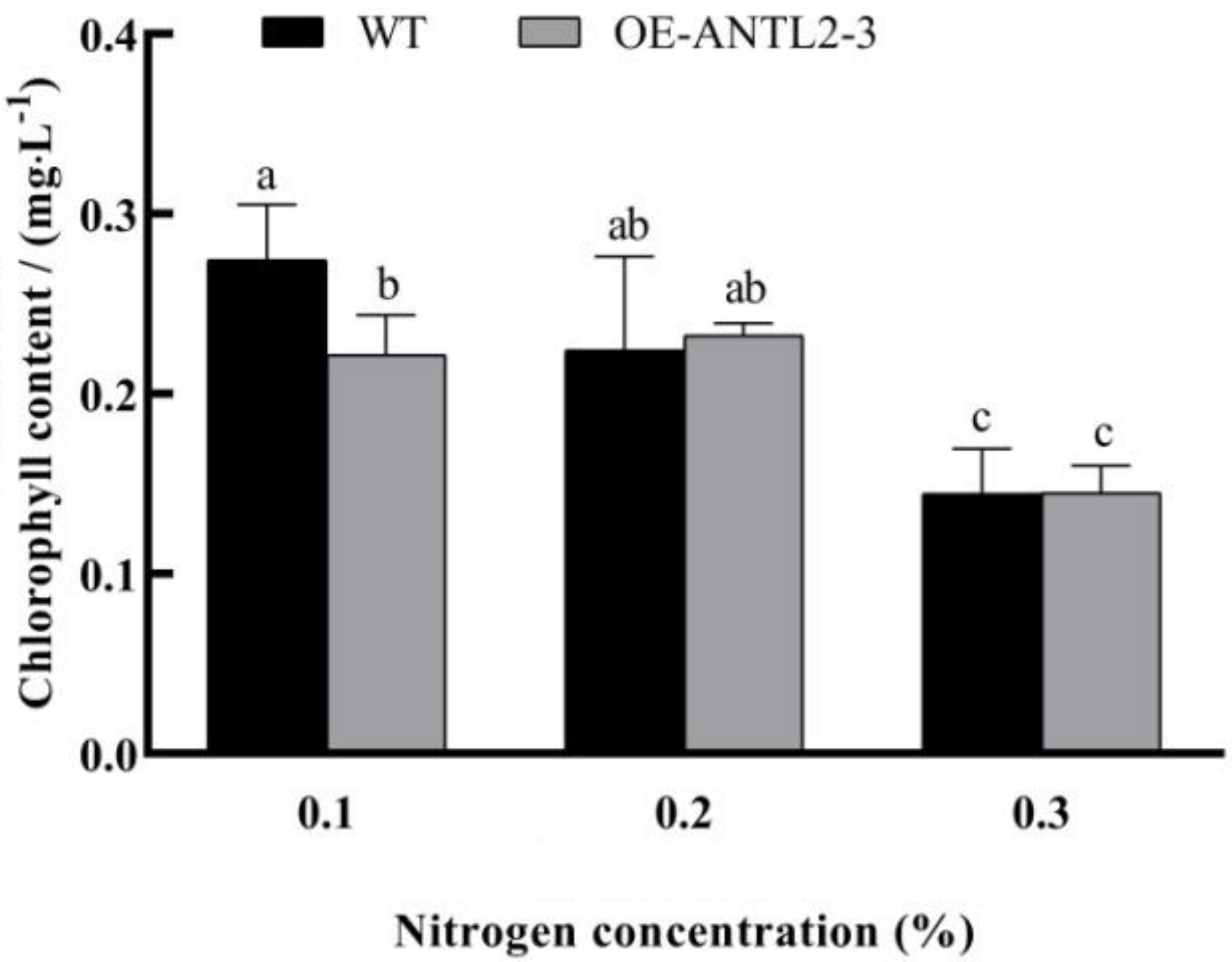

Fig. S3 Comparison of chlorophyll contents between transgenic plants and wild-type plants under different nitrogen levels

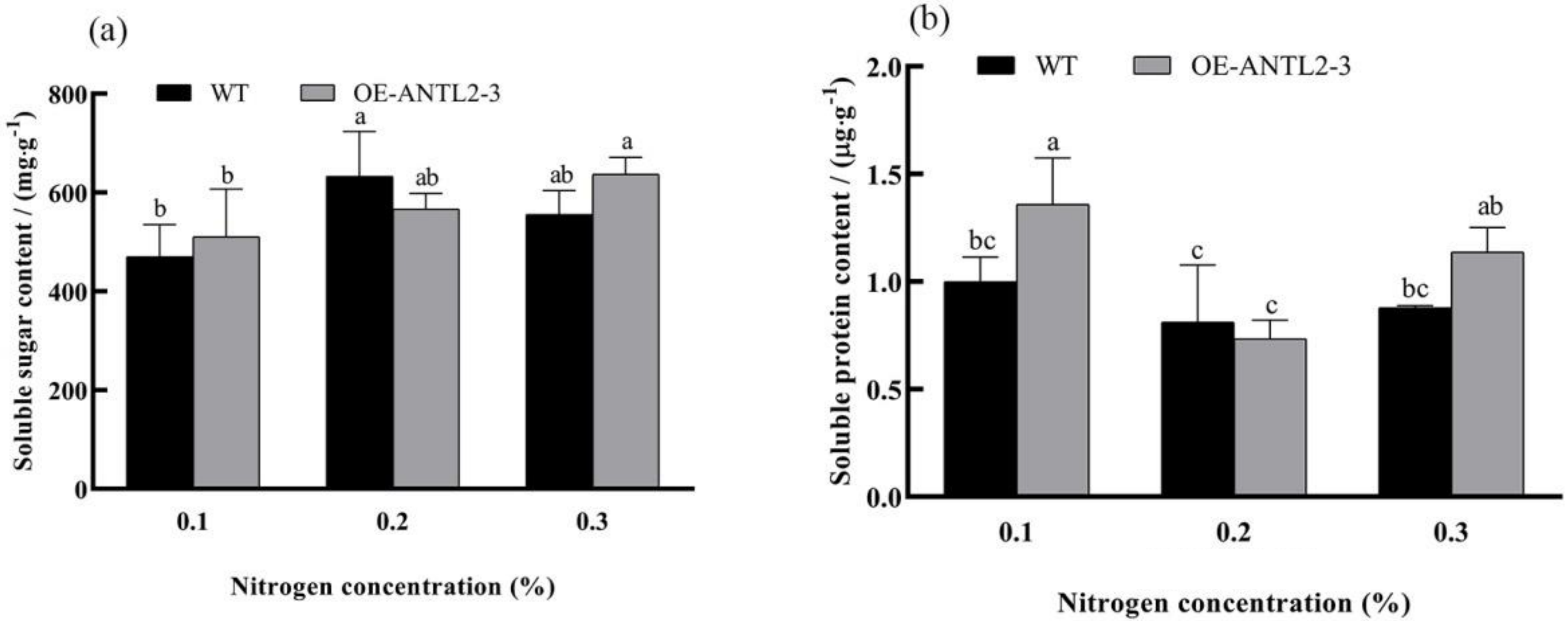

Fig. S4 Comparison of soluble sugar and soluble protein contents between transgenic plants and wild-type plants under different nitrogen levels

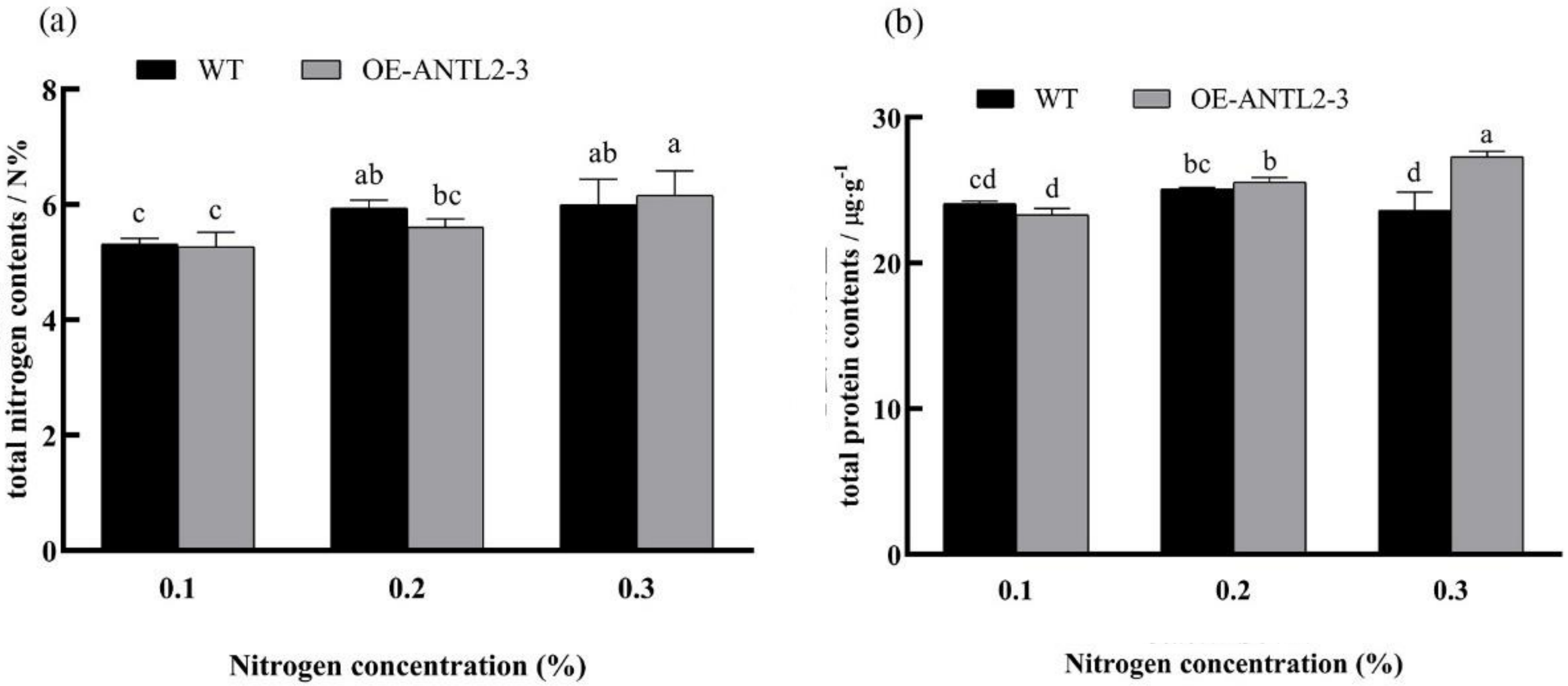

Fig.S5 Comparison of total nitrogen contents and total protein contents between transgenic plants and wild plants under different nitrogen levels
