# Supplementary material for: NtANTL2 overexpression regulates starch-related and nitrogen metabolism in tobacco plants
Source: BMC Plant Biol. 2025 Jun 7;25:775. doi: 10.1186/s12870-025-06748-8 (PMC12144802; doi:10.1186/s12870-025-06748-8)
Supplement: Supplementary file 2 — Supplementary Material 2: Supplementary Table 1. Primer names and sequences of clone NtANTL2. [file 12870_2025_6748_MOESM2_ESM.docx]

**Supplementary Table 1. Primers used in this study**

| *Gene* | Primer | Sequence (5’-3’) | Purpose |
| --- | --- | --- | --- |
| *NtANTL2-1* | QFNtANTL2-1 | TCACAACCAATCTTGGGAGAGG | qRT-PCR |
|  | QRNtANTL2-1 | GGTAGCACAAACCCCAAAACA | qRT-PCR |
| *NtANTL2-2* | QFNtANTL2-2 | TTTGGCTAAGATGGATTGTGG | qRT-PCR |
|  | QRNtANTL2-2 | GTCAAACCAAGCCATCCCAG | qRT-PCR |
| *NtANTL2-3* | QFNtANTL2-3 | CTGGAATCAGAGACAAGAGACAAGGAC | qRT-PCR |
|  | QRNtANTL2-3 | GCTAAGATCTCCATCAGGGAAGAAGAG | qRT-PCR |
| *NtANTL2-4* | QFNtANTL2-4 | GCAGGTATTGTCTTTGGATAAGATG | qRT-PCR |
|  | QRNtANTL2-4 | TAAGCCTTGGCTCCCAATATCTC | qRT-PCR |
| *NtANTL2-5* | QFNtANTL2-5 | TCACTTGTTGGGAGCAGTGTGTGC | qRT-PCR |
|  | QRNtANTL2-5 | CTAAGCCTTGGCTCCAAAG | qRT-PCR |
| *EF-1* | QEF-1F | TGAGATGCACCACGAAGCTC | qRT-PCR |
|  | QEF-1R | TGAGATGCACCACGAAGCTC | qRT-PCR |
| *NtANTL2-1* | RNtANTL2-1Z | ACCCGGGATGGGGTTTGAGAAAGACAAAGC | Clone |
|  | RNtANTL2-1Z | GGAGCTCTTATGCTTTGACTCCAAAGATCTC | Clone |
| *NtANTL2-2* | FNtANTL2-2Z | ACCCGGGATGGGGTTTGAGAAAGACAAGGC | Clone |
|  | RNtANTL2-2Z | GGAGCTCTCAAGCTTTGACTCCAAAGATC | Clone |
| *NtANTL2-3* | FNtANTL2-3Z | ACCCGGGATGGGGTTTGAGAAAGATGAAGC | Clone |
|  | RNtANTL2-3Z | GGAGCTCTTATGCCTTTTTTGCTAAGATCTC | Clone |
| *NtANTL2-4* | FNtANTL2-4Z | ACCCGGGATGGTGTTTGAGAAAAATAATAAAGC | Clone |
|  | RNtANTL2-4Z | GGAGCTCTTAAGCCTTGGCTCCCAATAT | Clone |
| *NtANTL2-5* | FNtANTL2-5Z | ACCCGGGATGGGGTTTGAGAAAAATAATAAAGC | Clone |
|  | RNtANTL2-5Z | GGAGCTCCTAAGCCTTGGCTCCAAAGAT | Clone |
